# Aloin Protects Skin Fibroblasts from Heat Stress-Induced Oxidative Stress Damage by Regulating the Oxidative Defense System

**DOI:** 10.1371/journal.pone.0143528

**Published:** 2015-12-04

**Authors:** Fu-Wei Liu, Fu-Chao Liu, Yu-Ren Wang, Hsin-I Tsai, Huang-Ping Yu

**Affiliations:** 1 Department of Physical Medicine and Rehabilitation, Taichung Veterans General Hospital Puli Branch, Nantou, Taiwan; 2 Department of Anesthesiology, Chang Gung Memorial Hospital, Taoyuan, Taiwan; 3 College of Medicine, Chang Gung University, Taoyuan, Taiwan; Imperial College London, Chelsea & Westminster Hospital, UNITED KINGDOM

## Abstract

Oxidative stress is commonly involved in the pathogenesis of skin damage induced by environmental factors, such as heat stress. Skin fibroblasts are responsible for the connective tissue regeneration and the skin recovery from injury. Aloin, a bioactive compound in Aloe vera, has been reported to have various pharmacological activities, such as anti-inflammatory effects. The aim of this study was to investigate the protective effect of aloin against heat stress-mediated oxidative stress in human skin fibroblast Hs68 cells. Hs68 cells were first incubated at 43°C for 30 min to mimic heat stress. The study was further examined if aloin has any effect on heat stress-induced oxidative stress. We found that aloin protected Hs68 cells against heat stress-induced damage, as assessed by 3-(4,5-dimethylthiazol-2-yl)-2,5-diphenyltetrazolium bromide and lactate dehydrogenase assay. Aloin protected Hs68 cells by regulating reactive oxygen species production and increasing the levels of glutathione, cytosolic and mitochondrial superoxide dismutase. Aloin also prevented the elevation of thiobarbituric acid reactive substances and the reduction of 8-OH-dG induced by heat stress. These results indicated that aloin protected human skin fibroblasts from heat stress-induced oxidative stress damage by regulating the oxidative defense system.

## Introduction

Being the largest organ of the body, skin is one of the first defense mechanisms of the immune system. Skin immunity allows skin itself to resist infections from pathogens and noxious stimuli. Skin ageing is a complex process and can be influenced by environmental factors such as hyperthermia [1]. Reactive oxygen species (ROS), such as superoxide anions, hydrogen peroxide and hydroxyl radical are chemically reactive molecules containing oxygen [2]. They are formed as natural byproducts of the normal oxygen metabolism and have important roles in cell signaling and homeostasis [3]. However, during times of environmental stress, ROS levels can increase dramatically [3]. At low levels, ROS regulate proliferation while at high levels; ROS are known to promote apoptosis [4]. ROS induce DNA and protein damages and enhance lipid peroxidation, leading to an increase in the production of malondialdehyde (MDA) as the end product [5], which may result in significant damage to cell structures [6]. Generally, under oxidative stress, skin defends itself against ROS-induced damage with enzymes such as superoxide dismutases (SODs), catalases, lactoperoxidases, glutathione peroxidases and peroxiredoxins [7,8]. Small-molecule antioxidants such as tocopherol, uric acid, ascorbic acid (vitamin C), and glutathione (GSH) also play important roles as cellular antioxidants [9,10].

Aloe vera plant has been widely used in alternative medicine, as health and nutritional supplements and also for cosmetic purposes. Aloe vera extracts, rich in polyphenols, have been shown to possess various pharmacological characteristics, including skin protective properties [11]. Other studies have also proposed that aloin (10-glucopyranosyl-1,8-dihydroxy-3- Hydroxylmethyl-9(10H)-anthracenone), a main bioactive compound in Aloe vera, to have anti-proliferative [12], anti-inflammatory [13] and anti-cancer properties [14]. However, the protective mechanisms of aloin in heat stress-induced skin damage is unclear.

In the present study, we evaluated the effects of aloin on the sensitivity of human skin fibroblast Hs68 cells to acute heat stress. We also aimed to investigate the mechanisms responsible for its effects, by performing 3-(4,5-dimethylthiazol-2-yl)-2,5-diphenyltetrazolium bromide (MTT) and lactate dehydrogenase (LDH) assays, and by measuring the levels of intracellular ROS, lipid peroxidation marker- MDA, oxidative DNA damage marker- 8-hydroxy-2’-deoxyguanosine (8-OH-dG), and oxidative defense molecules, such as GSH and SOD.

## Materials and Methods

### Materials

Aloin, hydrochloric acid, methanol, dimethyl sulfoxide (DMSO), doxycycline hyclate, calcium chloride (CaCl_2_), DL-dithiothreitol, and Folin-Ciocalteu reagent were purchased from Sigma-Aldrich Chemicals (St. Louis, MO, USA). Fetal bovine serum (FBS), penicillin-streptomycin, trypsin-EDTA, Dulbecco's Modified Eagle's Medium (DMEM) and Dihydrorhodamine-123 (DHR-123) were purchased from Gibco, Invitrogen (Carlsbad, CA, USA). Coomassie blue R-250, dibasic sodium phosphate, tris, and MTT were purchased from USB (Cleveland, OH, USA). All other chemicals used were of high purity biochemistry grade.

### Cell Culture

The human foreskin fibroblast Hs68 cells were purchased from the Cell Culture Center of the Food Industry Research and Development Institute (Hsinchu, Taiwan), and were cultured in Dulbecco’s modified Eagle medium (Life Technologies, Grand Island, NY, USA) in 75-cm^2^ (75-T) flasks with 10% fetal bovine serum (Equitech-Bio, Kerrville, TX, USA) at 37°C in a humidified incubator under 5% CO_2_ and 95% air. The cells, which had been passaged for 19 times (p19) before purchasing, were further sub-cultured in 75-T flasks continuously by trypsinization in 1:3 split, when the cell density reached confluence. The cells were amplified and stocked at passages of 27, 29 and 31 in liquid nitrogen for preservation. The survival rate of cells was higher than 95% by Trypan-blue assay [15].

### Heat Stress and Aloin Treatment

Heat stress was performed by immersing plastic vessels containing the attached cells in the water bath at 43°C (±0.05°C) for 30 min as previously described [16]. The temperature was monitored with a digital thermometer (No. 7563, Yokogawa, Tokyo) during heating. After heat stress treatment, Hs68 cells were seeded at 10^5^ cells/ml for various times (4–72 h) with aloin at 150 or 300 M as previously determined [17]. In non-induced culture, all the cells were cultured in the presence of the same doses of DMSO (vehicle < 0.5%). Then, all cells were incubated for 24–72 h at 37°C.

### Measurement of Cell Proliferation Using MTT Assay

Hs68 cells were cultured in the presence of DMSO or aloin for 24–72 h following heat stress treatment, and seeded into 96-well plates at a density of 10^5^ cells/cm^2^. Then the cells were incubated in medium containing 1.25 mg/ml of the MTT salt for 4 h at 37°C. The formazan product generated was solubilized by the addition of 20% sodium dodecyl sulfate and 50% N,N-dimethylformamide. Absorbance of the converted dye was recorded at 590 nm with a reference wavelength of 690 nm.

### Measurement of LDH Activity

The percentage of release of LDH was used as an index of cytotoxicity, and the activity was measured spectrophotometrically using pyruvate as substrate [18]. Total LDH was measured in cell lysates obtained by treatment with 0.5% Trition X-100, and the percentage of release was determined by dividing the LDH activity in the medium by total LDH activity.

### Measurement of Lipid Peroxidation Activity

Lipid peroxidation was measured as thiobarbituric acid-reactive substances (TBARS) released into the HBSS medium from Hs68 cells following centrifugation at 1,000 g for 10 min. TBARS were measured by mixing equal volumes of the supernatant with 0.7% TBA reagent and 2.5% trichloroacetic acid (TCA) [19]. Additional BHT (0.5 mM) was included to prevent sporadic lipid peroxidation during heating at 100°C for 10 min. TBARS were extracted with an equal volume (3 ml) of butanol and centrifuged briefly, and the fluorescence of the butanol layer was measured at 515 nm excitation and 555 nm emission [20]. The unit of TBARS was expressed as nmol MDA equivalent/10^6^ cells using 1,1,3,3-tetramethoxypropane as MDA standard.

### Detection of Intracellular ROS Using Fluorescence Assay

Hs68 cells were cultured in the presence of DMSO or aloin for 24–72 h following heat stress treatment, trypsinized, washed twice in PBS and then used for the determination of total ROS production using DHR-123 [21]. Intracellular ROS was analyzed by flow cytometry. Briefly, 10^5^ cells in PBS containing 0.9 mM CaCl_2_, 0.5 mM MgCl_2_, glucose (20 mM) and sodium azide (3 mM) were incubated for 15 min at 37°C in the dark in presence of DHR-123 at 10 μM. Finally, the cells were washed and kept on ice until flow cytometry analysis. Total ROS productions were expressed as mean fluorescence intensity (MFI) in arbitrary units (AU) as determined using Cell Quest software (BD biosciences).

### Determination of 8-OH-dG Concentration

Oxidative DNA damage was assessed by measuring 8-OH-dG concentration with a competitive immunoassay (Cell Biolabs Inc., San Diego, CA, USA). In brief, the cells were cultured in Lab-Tek Chamber Slides and exposed to DMSO or aloin for 24–72 h following heat stress treatment, and then a total genomic DNA was extracted from Hs68 cells using a GenElute Mammalian Genomic DNA Miniprep Kit (Sigma). DNA samples containing unknown concentrations of 8-OH-dG were then mixed with 8-OH-dG standards and incubated with an anti-8-OH-dG antibody for 1 h. Bound antibodies were detected after the incubation with horseradish peroxidase-labeled secondary antibody, and 8-OH-dG concentrations were read from the standard curve and expressed as picogram of 8-OH-dG per 10^5^ cells.

### Measurements of the Total GSH Levels

Total GSH levels were determined using a total glutathione quantitation kit, according to the instruction manual (Dojindo molecular technologies, Japan). Briefly, cells were seeded on a 10 cm dish and pretreated with heat stress challenge at 43°C for 30 min. Then, they were incubated with 150 μM or 300 μM aloin or the same dose of DMSO for 24–72 h. The treated cells were lysed in 10 mM hydrochloric acid solution by freezing and thawing. Then, they were treated with 5% 5-sulfosalicyclic acid. After centrifugation at 8000 x g for 10 min at 4°C, the supernatant was used to assess the GSH levels. The absorbance was measured using a spectrophotometer at a wavelength of 405 nm, and the GSH concentrations were determined using a GSH standard calibration curve.

### Measurement of Total SOD Activity

Total SOD activity was measured in cell lysates with a SOD assay kit (Cayman, An Arbor, MI, USA), as per the manufacturer's instructions. In the assay, superoxide ions were generated by the xanthine/xanthine oxidase system and convert nitroblue-tetrazolium (NBT) to NBT-diformazan, which was detected by light absorption at 560 nm. By neutralizing superoxide, SOD decreased the rate of NBT conversion in proportion to SOD activity in a sample. The results were expressed as milliunits of SOD activity per 10^5^ cells.

### Statistical Analysis

Results are expressed as means ± SEM. for the number of experiments indicated. All statistical analysis of data was computed using StatView (SAS Institute, CA). The sources of variation for multiple comparisons were assessed by ANOVA. The differences were considered statistically significant at P < 0.05.

## Results

### Aloin Improved Heat Stress-Induced Cell Viability and Proliferation

**Fig 1:**
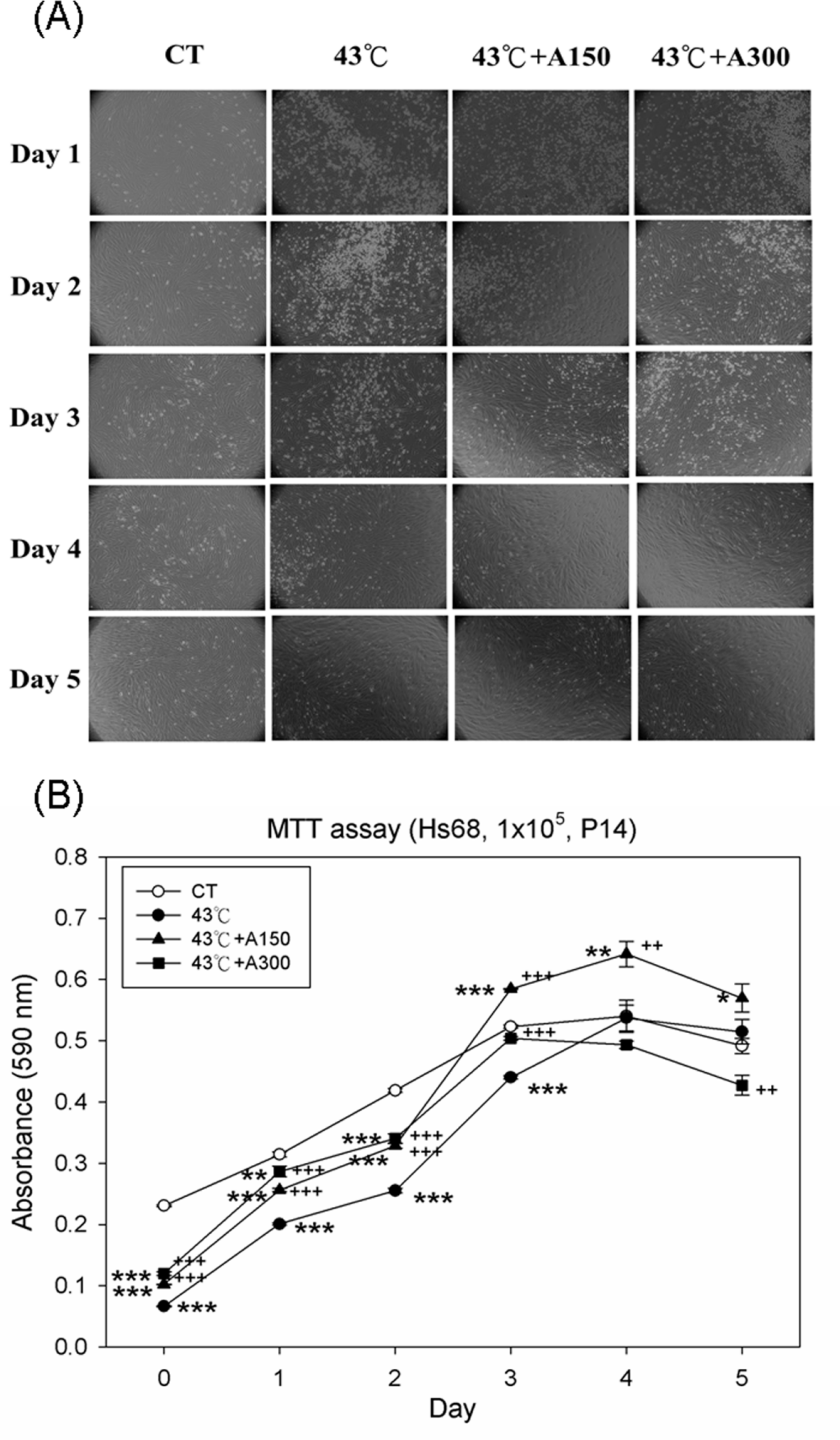
Effect of Aloin on cell viability of human foreskin fibroblast Hs68 cells exposed to 43°Cwith/without Aloin supplement. (A) The optical microscopy images (magnification, ×100) of fibroblast cell and (B) diagram of curve of cell viabilities were representative of three independent experiments. Cells were exposed to heat stress with a single administration of Aloin (43°C+A150) or (43°C+A300), vehicle (43°C), or were left unheated and given the vehicle (CT). The cell viabilities were measured by 3-(4,5-dimethylthiazol-2-yl)-2,5-diphenyltetrazolium bromide assay. The data are expressed as the means ± SEM of two replicates of three samples (n = 6). The asterisks indicate a significant difference (* P<0.05, ** P<0.01, *** P<0.005) compared to the control group; the crosses indicate a significant difference (^**++**^ P<0.01, ^**+++**^ P<0.005) compared to the heat-treated group.

In order to examine the effect of aloin (ranging from 150 to 300 μM) on cell viability *in vitro*, the MTT assay was performed on Hs68 cultures exposed to 43°C for 30 min. The results showed the decreased Hs68 cell viability and proliferation following 43°C heat treatment in a time-dependent manner. The optical image showed that the fibroblast cell proliferative activity was decreased following heat stress, and was partly improved by aloin (150 or 300 μM) from day 1 to day 3. Conversely, there was no significant difference of fibroblast proliferation in all groups at day 4 and day 5 (Fig 1A). Treatment with 150 or 300 μM aloin significantly increased cell viability compared to only 43°C-treated cells during day 0 to day 3 (Fig 1B).

### Aloin Reduced Heat Stress-Induced Cytotoxicity and Cell Morphology Change

**Fig 2:**
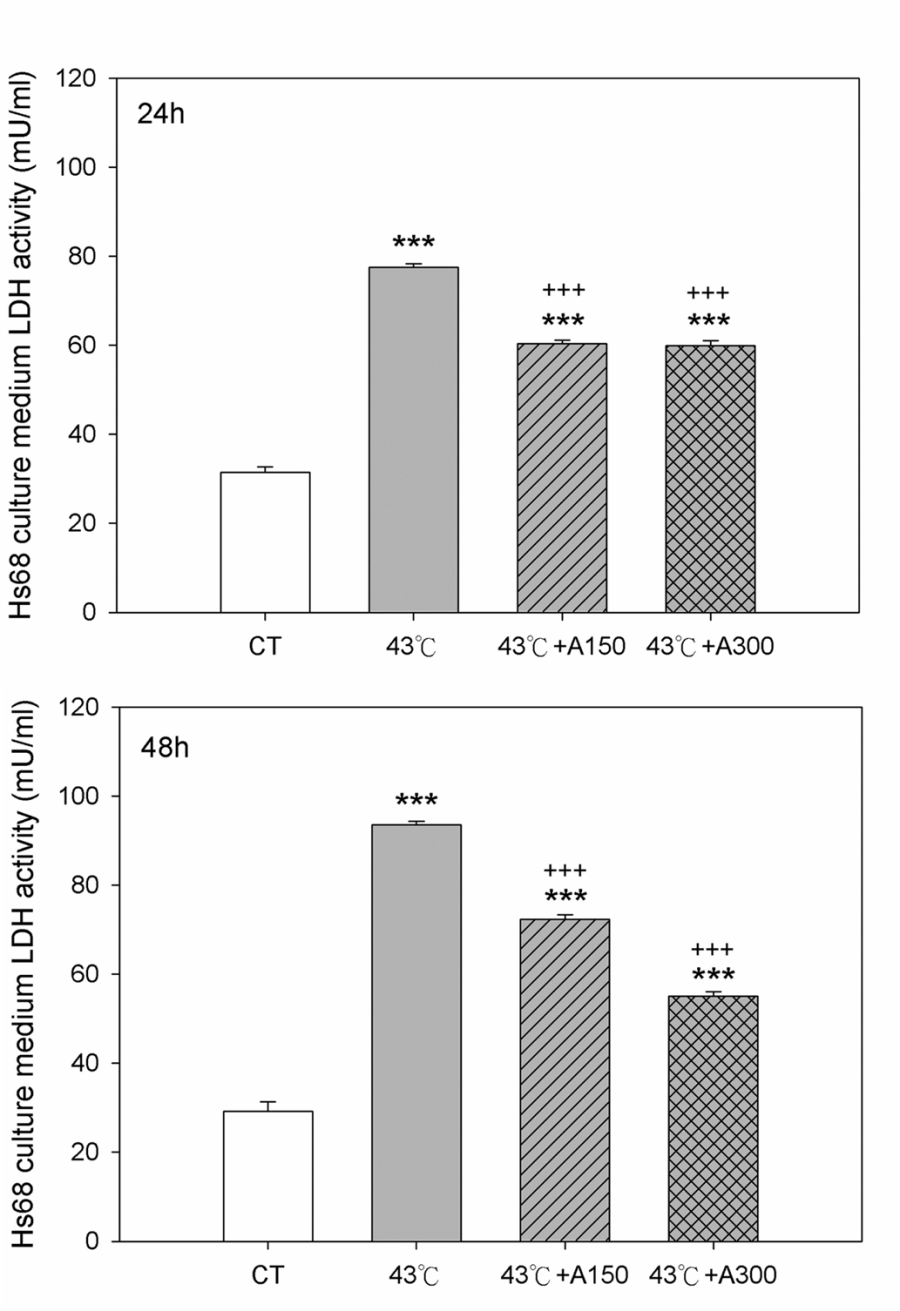
Effect of Aloin on lactate dehydrogenase (LDH) release in Hs68 cells with/without heat stress challenge. Cells were exposed to heat stress with a single administration of Aloin (43°C+A150) or (43°C+A300), vehicle (43°C), or were left unheated and given the vehicle (CT). The LDH activities were measured by ELISA. The data are expressed as the means ± SEM of two replicates of three samples (n = 6). The asterisks indicate a significant difference (*** P<0.005) compared to the control group; the crosses indicate a significant difference (^**+++**^ P<0.005) compared to the heat-treated group.

Cell morphology examination and LDH assay were performed to explore whether aloin affected the heat stress-induced cell morphology change and the cytotoxicity. Cell morphology markedly changed when the cells were incubated at 43°C for 30 min, but were partly reversed by aloin (150 or 300 μM) up to 72 h (data not shown). The activities of LDH released into the medium were significantly increased by heat stress challenge in Hs68 cells and LDH activities were significantly attenuated at 24 hr or 48 h with aloin (150 or 300 μM) treatment. (Fig 2). Enrichment of the non-heat challenge cells with aloin had no effect on cell morphology (data not shown).

### Aloin Reduced Heat Stress-Mediated Lipid Peroxidation

To further characterize the antioxidant effect of aloin, we then used 150 or 300 μM aloin to study the time course of lipid peorxidation induced by heat stress challenge. Table 1 shows the effect of aloin on heat stress-mediated lipid peroxidation in Hs68 cells. In Hs68 cells incubated at 43°C for 30 min, TBARS increased (from mean 6.1± 0.3 to 11.2±0.9, 6.3±0.5 to 12.5± 1.2 and 6.4±0.7 to 11.9±0.8 nmol MDA/10^6^ cells, respectively) at 24 to 72 h (P<0.01). However, in cells treated with aloin (150 or 300 μM), the activities of TBARS were significantly attenuated at 24–72 h in a dose-dependent manner (Table 1).

**Table 1:** Activity of TBARS in Hs68 cells following heat stress and various concentration aloin treatment.

Treatment	TBARS (nmol MDA equivalent/10^6^ cells)		
	24 h	48 h	72 h
CT	6.1 ± 0.3	6.3 ± 0.5	6.4 ± 0.7
43°C	11.2 ± 0.9 **	12.5 ± 1.2 **	11.9 ± 0.8 **
43°C+A150	8.6 ± 0.5	7.3 ± 0.5 ^**++**^	7.1 ± 0.6 ^**++**^
43°C+A300	8.4 ± 0.6 ^**+**^	6.1 ± 0.3 ^**+++**^	5.8 ± 0.4 ^**+++**^

Cells were exposed to heat stress with a single administration of Aloin (43°C+A150) or (43°C+A300), vehicle (43°C), or were left unheated and given the vehicle (CT). The data are expressed as the means ± SE. of duplicates. The asterisks indicate a significant difference (**: P<0.01) compared to the control group; the crosses indicate a significant difference (+++: P<0.005, ++: P<0.01, +: P<0.05) compared to the heat-treated group.

TBARS: thiobarbituric acid-reactive substances; Hs68 cells:human foreskin fibroblasts; MDA: malondialdehyde.

**: P<0.01

+++: P<0.005

++: P<0.01

+: P<0.05)

### Aloin Attenuated Heat Stress-Induced Intracellular ROS Production

**Fig 3:**
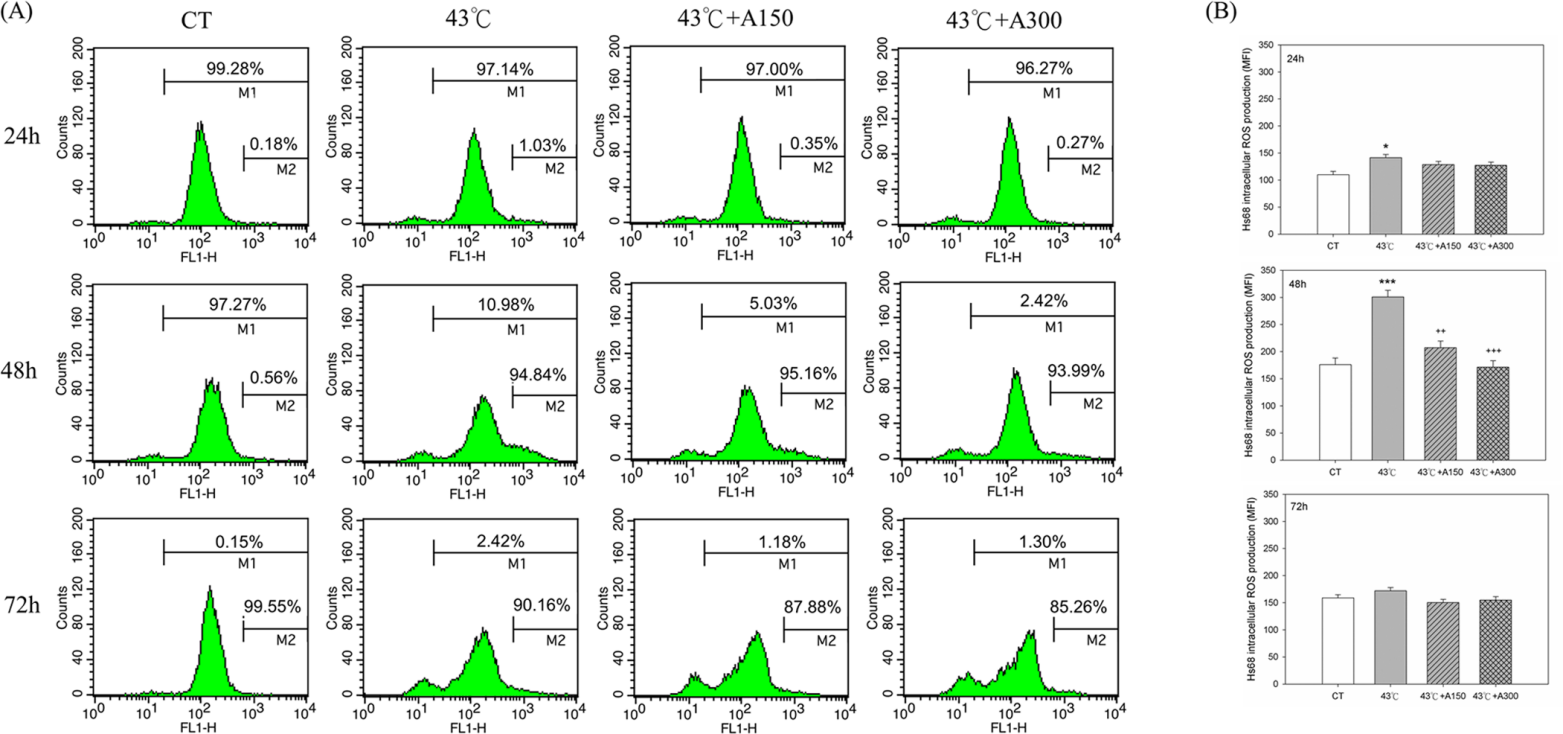
Effect of Aloin on intracellular ROS release in Hs68 cells with/without heat stress challenge. Cells were exposed to heat stress with a single administration of Aloin (43°C+A150) or (43°C+A300), vehicle (43°C), or were left unheated and given the vehicle (CT). Representative staining of ROS in HS68 cultures probed with DHR-123 by flow cytometry analysis (A). The data are expressed as the means ± SEM of two replicates of three samples (n = 6) (B). The asterisks indicate a significant difference (* P<0.05, *** P<0.005) compared to the control group; the crosses indicate a significant difference (^**++**^ P<0.01, ^**+++**^ P<0.005) compared to the heat-treated group. Experiments were performed in duplicates with Hs68 cultures derived from 5 different donors.

To examine whether the protective effect of aloin against heat stress-induced cell toxicity was related to antioxidant effects, intracellular ROS generation using DHR-123 was assayed. The experiments on cells continuously exposed to 43°C for 30 min and then probed with a fluorescent dye, DHR-123, showed that the generation of ROS at 24 and 48 h by Hs68 cells was higher (29±3% and 43±5%) as compared to CT group (P<0.05) (Fig 3). An even stronger inhibiting effect was observed in cells exposed to 150 or 300 μM aloin at 48 h and a decrease by 31±5% and 92±39% (P<0.01) compared to 43°C group. However, the production of ROS in cells treated with 150 or 300 μM aloin had no inhibiting effect at 24 or 72 h compared to 43°C group.

### Aloin Decreased Heat Stress-Induced DNA Damage

**Fig 4:**
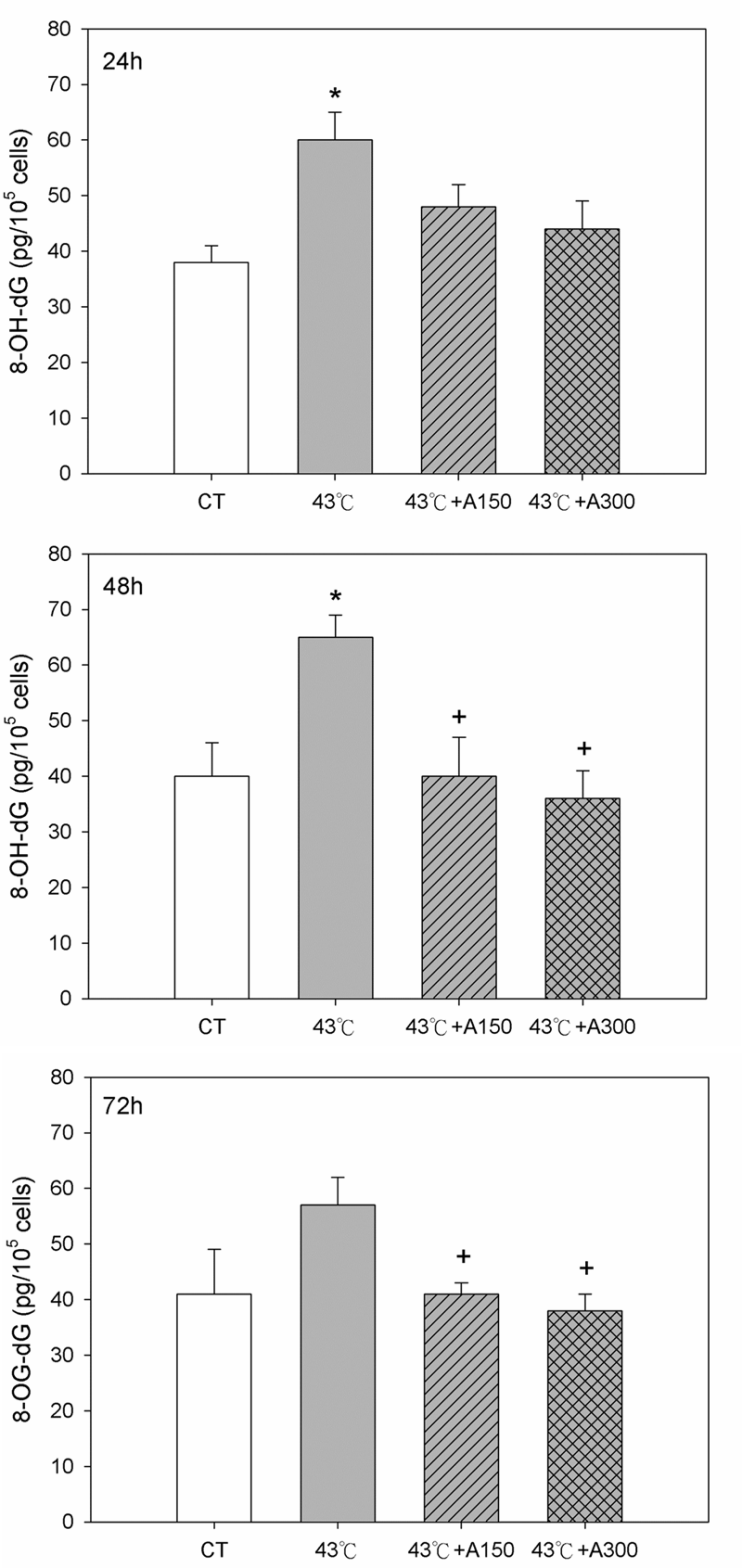
Effect of Aloin on 8-OH-dG concentration in Hs68 cells with/without heat stress challenge. Cells were exposed to heat stress with a single administration of Aloin (43°C+A150) or (43°C+A300), vehicle (43°C), or were left unheated and given the vehicle (CT). The 8-OH-dG levels were measured by ELISA. The data are expressed as the means ± SEM of two replicates of three samples (n = 6). The asterisks indicate a significant difference (* P<0.05) compared to the control group; the crosses indicate a significant difference (^**+**^ P<0.05) compared to the heat-treated group.

Next, the magnitude of DNA injury in Hs68 cells propagated in the presence of heat stress was examined according to the measurements of the concentration of the oxidative DNA adduct, 8-OH-dG. Studies of 8-OH-dG level showed that after 30 min of exposure to 43°C, the concentration of this DNA adduct in Hs68 cells at 24–48 h was increased by 58±5% and 63±6% higher (P<0.05) compared to control cells (Fig 4). Noteworthy, the concentration of 8-OH-dG in cells exposed to 150 μM aloin at 48–72 h was significantly decreased by 38±8% and 28±3% lower than in cells exposed to 43°C group (P<0.05). As well as 150 μM aloin, the 8-OH-dG levels in Hs68 cells at 48–72 h exposed to 300 μM aloin were also lower (P<0.05) than in the heat-treated cells.

### Aloin Up-Regulated Heat Stress-Mediated GSH Production and SOD Activity

**Fig 5:**
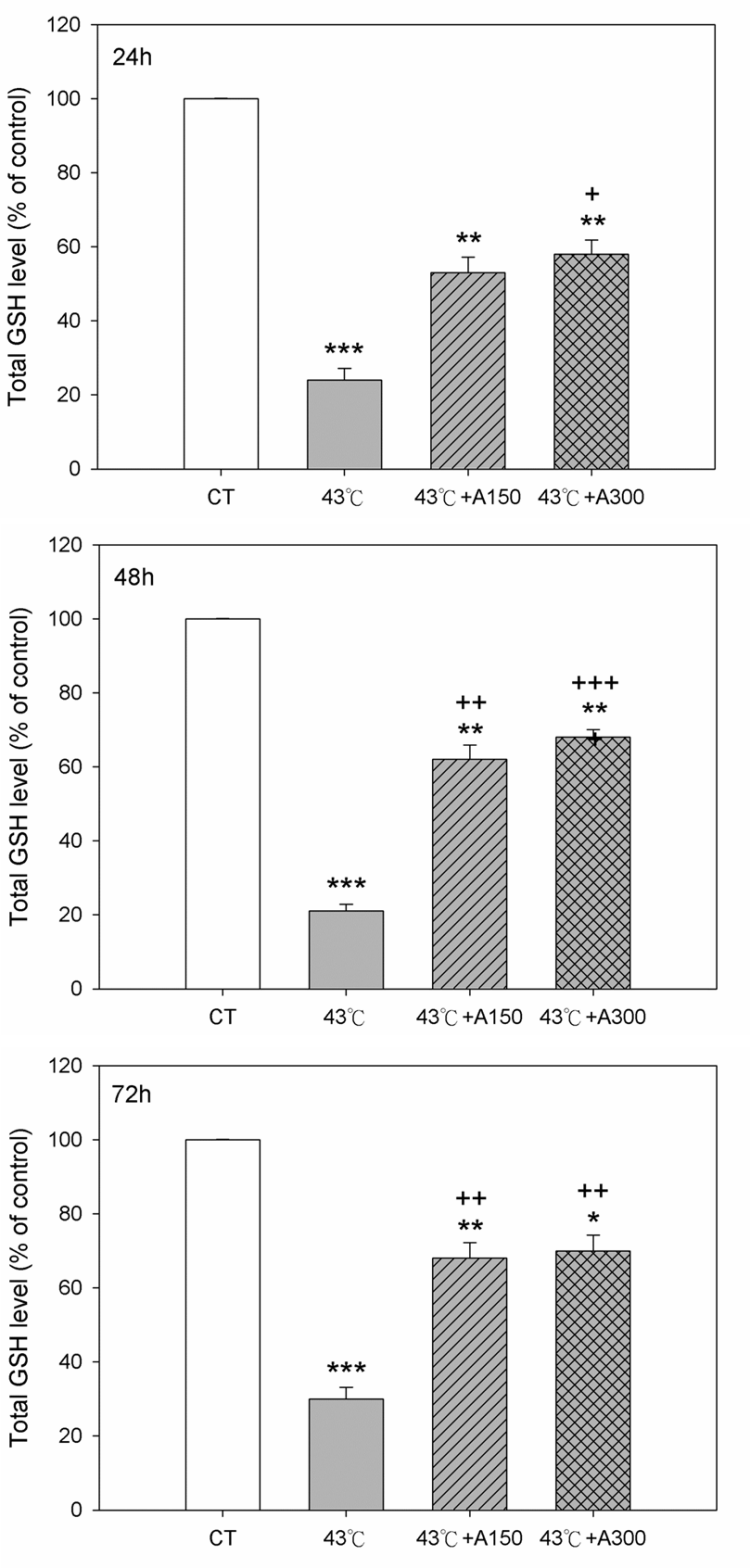
Effect of Aloin on total GSH levels in Hs68 cells with/without heat stress challenge. Cells were exposed to heat stress with a single administration of Aloin (43°C+A150) or (43°C+A300), vehicle (43°C), or were left unheated and given the vehicle (CT). The GSH levels were measured by ELISA. The data are expressed as the means ± SEM of two replicates of three samples (n = 6). The asterisks indicate a significant difference (* P<0.05, ** P<0.01, *** P<0.005) compared to the control group; the crosses indicate a significant difference (^**+**^ P<0.05, ^**++**^ P<0.01, ^**+++**^ P<0.005) compared to the heat-treated group.

To examine the relation between GSH and heat stress-mediated oxidative stress, changes in intracellular GSH were determined 30 min after 43°C exposure. As shown in Fig 5, 43°C caused a significant depletion (P<0.001) in intracellular GSH levels at 24–72 h, as compared to that of non-heated cells. Treatment with aloin at concentrations of 150 and 300 μM at 24–72 h effectively elevated the GSH levels by 2.4-fold and 2.6-fold, respectively.

**Fig 6:**
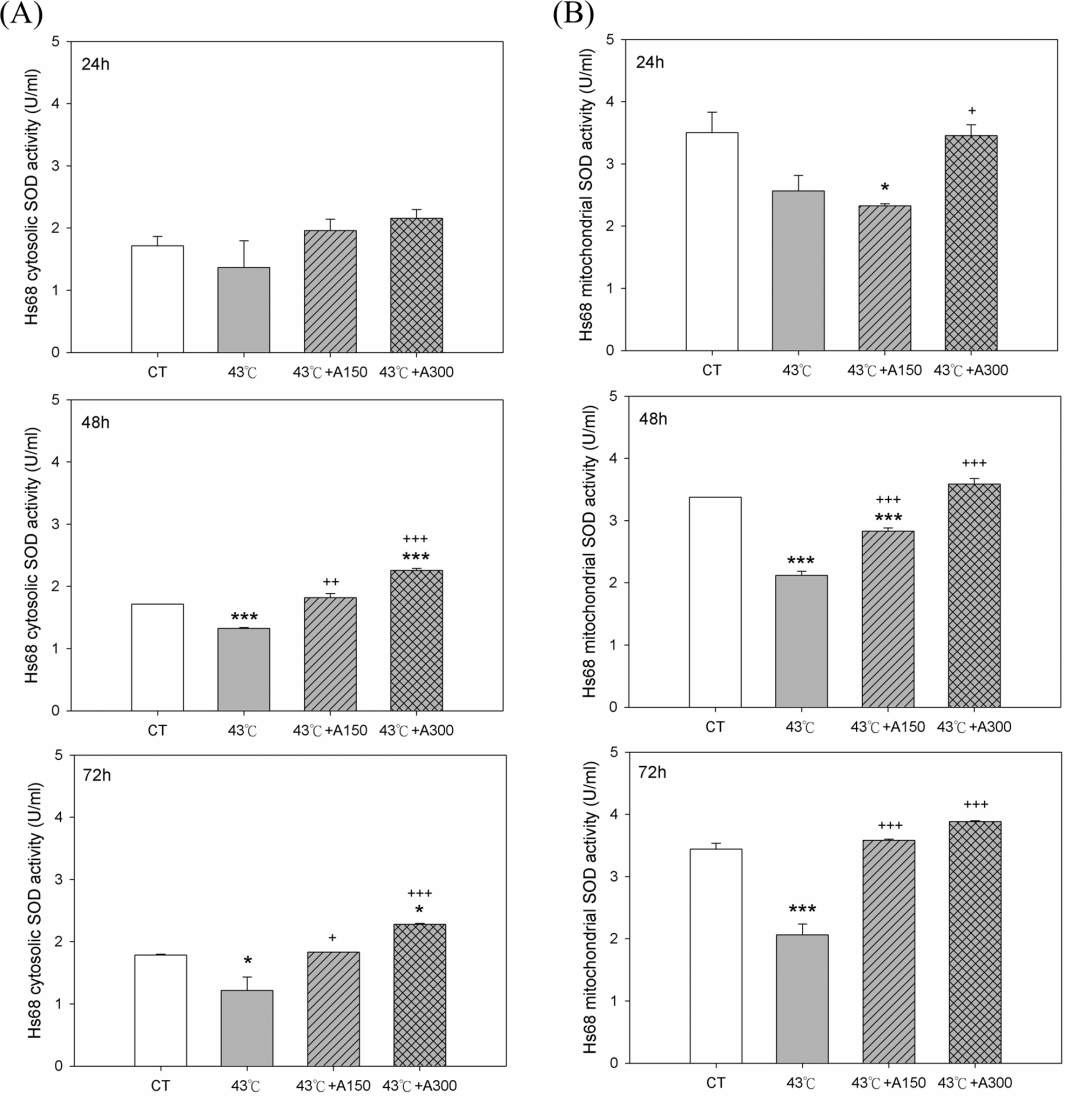
Effect of Aloin on (A) cytosolic and (B) mitochondrial SOD activities in Hs68 cells with/without heat stress challenge. Cells were exposed to heat stress with a single administration of Aloin (43°C+A150) or (43°C+A300), vehicle (43°C), or were left unheated and given the vehicle (CT). The cytosolic and mitochondrial SOD activities were assayed by ELISA. The data are expressed as the means ± SEM of two replicates of three samples (n = 6). The asterisks indicate a significant difference (* P<0.05, *** P<0.005) compared to the control group; the crosses indicate a significant difference (^**++**^ P<0.01, ^**+++**^ P<0.005) compared to the heat-treated group.

To investigate whether aloin affected the heat stress-regulatory antioxidant enzyme system, the total activity of SOD was used as a measure of the anti-oxidative cell capacity. The measurements performed using the commercial xanthine/xanthine oxidase system showed that cytosolic SOD activity in Hs68 cells exposed to 43°C at 48–72 h was lower by 23±2% and 32±5% (P<0.05) as compared to the control ones (Fig 6A). On the other hand, the Hs68 cells exposed to 150 μM aloin displayed cytosolic SOD activity, at 48–72 h, was higher by 37±5% and 50±2% (P<0.05) compared to the 43°C group. The enzyme activity in Hs68 cells treated with 300 μM aloin was significantly greater than in cells treated with 150 μM aloin (P<0.001). As similar as cytosolic SOD activity pattern, the mitochondrial SOD levels in Hs68 cells exposed to 150 or 300 μM aloin at 48–72 h were also higher (P<0.05) than in the heat-treated cells (Fig 6B).

## Discussion

The results of the present study demonstrate that aloin reduces heat stress-induced ROS overproduction and oxidative damage to lipid and DNA in human fibroblast Hs68 cells. The potential mechanism may be mediated by the enhancement of oxidative defense system, including GSH and SOD activities. These overall observations illustrate that aloin may protect human skin fibroblasts against heat stress-induced oxidative damage.

The accumulation of ROS, encompassing the amount necessary to explicate physiological signaling functions, is implicated as a mediator of a variety of human diseases inducing DNA damage, lipid peroxidation, and protein disaggregation, as well as increasing peroxinitrite and other reactive nitrogen species [22,23]. To prevent and/or to counteract oxidative damage, all aerobic organisms use a lot of enzymatic and non-enzymatic systems, including transitory or permanent growth arrest, apoptosis, or necrosis, depending on the severity of the stress. Oxidative stress-induced apoptosis, known to be associated with falls in GSH levels [24,25] as well as with rises in carbonyls and lipid peroxidation with subsequent membrane breakdown, has already been reported to be co-related [26,27].

Previous literatures have indicated that ROS may potentially induce skin aging-related proteins and cause intracellular oxidative damage in human skin fibroblasts [28]. Furthermore, gene networks can be related to cell death elicited by heat stress in mild hyperthermic conditions and hyperthermia at relatively high temperature [29,30] in normal cells and cancer cells [31,32]. Lin et al. reported that hyperthermia stimulated the reaction of purine and xanthine oxidase, leading to an increased cytotoxic effect of reactive oxygen species to Chinese hamster cells and bovine endothelial cells in vitro [33]. In this study, we demonstrated that an overproduction of ROS that occurred in human skin fibroblast Hs68 cells after exposure to heat stress contributed to increased levels of TBARS and 8-OH-dG, thereby reducing Hs68 cell viability.

Additionally, it was reported that treatment with Aloe vera increased antioxidant enzymes and drastically reduced lipid peroxidation products [34]. The phenomenon Aloe vera extract caused a reduction of the MDA levels suggested that pro-oxidative damage might decrease with the administration of Aloe vera extracts of different growing stages of the plant, demonstrating a strong inhibition activity on lipid oxidation [35]. Yagi et al. reported that isorabaichromone, a chromone components from Aloe vera, showed antioxidative activity against microsomal and mitochondrial non-enzymatic lipid peroxidation [36].

In our study, a markedly increased production of total ROS and 8-OH-dG were detected in heat stress-treated cells. Moreover, aloin was found to effectively induce less total ROS production in Hs68 cells exposed to heat stress as determined by using DHR-123. However, DHR, which passively diffused across membranes and oxidize to cationic rhodamine-123 were found to localize in the mitochondria [37], suggesting that the intracellular retention of rhodamine-123 was dependent on the integrity of the mitochondrial membrane. Therefore, the increase of the mitochondrial SOD activity induced by aloin might explain the low ROS production.

In response to ROS production, a cell antioxidant response via enzymatic activities may be expected. However, aloin significantly increased activities of GSH and total SOD levels under heat stress condition in both cytosol and mitochondria. This suggests that Hs68 cells, in response to ROS attack, were able to regulate the antioxidant system at the post-transcriptional level [38]. Moreover, suppressed ROS generation may directly enhance the enzymatic activity, explaining why GSH and total SOD levels were increased after aloin treatment. All of which implied that the nature and the impact of ROS attack, providing a possible mechanism against heat stress-induced damage in skin fibroblasts.

Dermal fibroblasts are major cells within the skin dermis layer which are responsible for damage connective tissue regeneration and skin healing [39]. Aloe Vera can enhance the penetration of molecules through skin epidermal layer [40,41]. Aloin is a bioactive extract of Aloe Vera [13]. Our results showed that Aloin reduce heat stress-induced fibroblasts injury in vitro. It is implied that sufficient concentration of Aloin may reach the target of subcutaneous fibroblast. However, further studies remain to be determined.

## Conclusion

In conclusion, aloin protected against heat stress -induced skin cell damage by reducing ROS overproduction and ameliorating the dissipation of oxidative defense system such as GSH and total SOD. Consequently, aloin could be promising in the design and development of possible treatment strategies aimed at limiting heat stress-induced cellular oxidative damage.

## Supporting Information

S1 File Numerical data of cell viability in human fibroblast Hs68 cells. (XLS)

S2 File Numerical data of lactate dehydrogenase release in human fibroblast Hs68 cells. (XLS)

S3 File Numerical data of intracellular reactive oxygen species release and flow cytometry code in human fibroblast Hs68 cells. (XLS)

S4 File Numerical data of 8-OH-dG concentration in human fibroblast Hs68 cells. (XLS)

S5 File Numerical data of glutathione levels in human fibroblast Hs68 cells. (XLS)

S6 File Numerical data of cytosolic and mitochondrial superoxide dismutase activities in human fibroblast Hs68 cells. (XLS)
